# Dietary approach to stop hypertension (DASH), but not Mediterranean and MIND, dietary pattern protects against Parkinson's disease

**DOI:** 10.1002/fsn3.3809

**Published:** 2023-11-06

**Authors:** Majid Keramati, Sorayya Kheirouri, Masoud Etemadifar

**Affiliations:** ^1^ Faculty of Nutrition and Food Sciences Tabriz University of medical sciences Tabriz Iran; ^2^ Student Research Committee Tabriz University of Medical Sciences Tabriz Iran; ^3^ Department of Neurosurgery Isfahan University of Medical Sciences Isfahan Iran

**Keywords:** DASH, dietary pattern, disease severity, MeDi, MIND, Parkinson's disease

## Abstract

The neuroprotective effects of dietary patterns have been reported in previous studies. This study aimed to examine the association between the dietary approach to stop hypertension (DASH), the Mediterranean diet (MeDi), and the Mediterranean‐DASH intervention for neurodegenerative delay (MIND) with the severity and risk of Parkinson's disease (PD). In this comparative cross‐sectional study, 120 patients with PD and 50 healthy participants participated. Adherence to DASH, MeDi, and MIND dietary patterns was determined according to the dietary intake data using a food frequency questionnaire (FFQ). The Severity of PD was determined by the Unified Parkinson's Disease Rating Scale (UPDRS). The mean score of the DASH was significantly lower in the PD group compared to the healthy group (*p* = .006), but the mean score of MeDi and MIND did not significantly differ between the two groups (*p* > .05). Also, the mean score of the DASH was significantly lower in men than in women in the healthy group (*p* = .018). High adherence to the DASH diet decreased the risk of PD by 15% (OR = 0.856, 95% CI: 0.751, 0.976, *p* = .020). Participants in quartiles 3 and 4 of the DASH dietary pattern had 86% (*p* = .003) and 87% (*p* = .007), respectively, lower risk of PD. MeDi and MIND diets were not significantly associated with the risk of PD. There was no significant association between dietary patterns and the severity of PD. The findings indicate that high adherence to the DASH dietary pattern may protect against PD.

## Graphical abstract

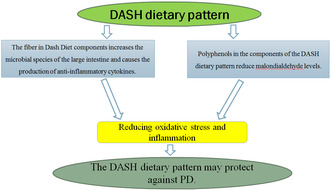

## 1 INTRODUCTION

Parkinson's disease (PD) is a neurological ailment affecting millions of people worldwide (Marras et al., 2018). The loss of dopaminergic neurons in the substantia nigra is a hallmark of PD. However, the condition is characterized by a larger dysfunction in other parts of the brain, including non‐dopaminergic neurons (Sung & Nicholas, 2013). According to research on the global burden of disease, 6.2 million people have PD, and this number is expected to double by 2040 (Brain, 2007; Noyes et al., 2006). By the age of 85, it is estimated that half of all older people will have developed symptoms of PD, which are often progressive and associated with disability (Buchman et al., 2016). Debilitating features of PD are bradykinesia, anxiety, tremor, cognitive impairments, and depression (Ayano, 2016; Hallett, 2012).

The exact pathogenic pathways of Parkinson's disease are unknown, but hereditary and environmental variables may play a role in disease pathology (De Lau & Breteler, 2006). The positive impact of lifestyle variables such as a healthy diet on the disease is encouraging, and researchers have observed the potential involvement of nutrition in the prevention, control, and recurrence of the disease (Ascherio et al., 2001; Chen et al., 2003, 2007; Rothenberg, 2019; Zhang et al., 2014). Some nutrients with anti‐inflammatory and antioxidant properties, including vitamin E, vitamin C, and carotenoids, may be useful in reducing the risk of PD (Agarwal et al., 2018).

Nutritional studies currently focus on the synergistic effects of nutrients rather than specific nutrients or diets in maintaining optimum health (Abbatecola et al., 2018; Boulos et al., 2019; Fernández et al., 2019; Pistollato et al., 2018; Solfrizzi et al., 2017). Some dietary patterns have been shown to affect a range of non‐communicable diseases (Appel et al., 2006; Ard et al., 2004; Bulló et al., 2011). These factors and conditions, such as hypertension and diabetes, cause an increased incidence of cognitive diseases and PD (Gorelick, 2010; Kivipelto et al., 2005; Saczynski et al., 2008). The dietary approach to stop hypertension (DASH) is a dietary pattern that recommends the consumption of fruits, vegetables, and low‐fat dairy. It also includes whole grains, poultry, fish, and nuts and reduces the consumption of red meat, sweets, sweet drinks, total fat, and cholesterol. Therefore, the DASH dietary pattern causes enhanced use of protective nutrients for non‐communicable diseases such as K, Ca, Mg, fiber, and plant proteins while decreasing the intake of refined carbohydrates, saturated fat, and sodium (Couch et al., 2021). According to the literature, adhering to the DASH dietary pattern improves brain function and reduces the incidence of cognitive diseases (Tangney et al., 2014).

The Mediterranean diet (MeDi) is a nutritional pattern popular in Mediterranean nations such as Greece (Trichopoulou et al., 2014). The MeDi is known for its high intake of fruits, vegetables, legumes, and whole grains. This dietary pattern also suggests a high intake of fish and unsaturated fatty acids, a low to moderate intake of dairy products, and a low intake of saturated fatty acids, meat, and poultry (Trichopoulou et al., 2003). An inverse relationship between MeDi and various non‐communicable diseases has recently been discovered (Scarmeas et al., 2006; Singh et al., 2002; Trichopoulou et al., 2000). Furthermore, MeDi has been demonstrated to increase brain function and lower the risk of Parkinson's disease (Agarwal et al., 2018; Scarmeas et al., 2006; Tangney et al., 2014; Trichopoulou et al., 2003, 2014; Yin et al., 2021). Two studies showed a reduced risk of prodromal PD (Maraki et al., 2019; Molsberry et al., 2020).

Another dietary pattern that is useful for the prevention of the initiation and progression of cognitive diseases and PD is the Mediterranean‐DASH intervention for neurodegenerative delay (MIND). A MIND diet has been developed for the prevention of cognitive diseases (Agarwal et al., 2018). This dietary pattern is a hybrid of the DASH and MeDi diets, and it is based on food compounds that have been identified as having neuroprotective properties. The MIND diet emphasizes the consumption of natural plant foods and the limited consumption of animal foods and foods rich in saturated fats. In particular, the MIND diet is known for its consumption of berries and green leafy vegetables (Morris, Tangney, Wang, Sacks, Barnes, et al., 2015; Morris, Tangney, Wang, Sacks, Bennett, & Aggarwal, 2015). According to reports, long‐term adherence to the MIND diet improves brain function (Berendsen et al., 2018). Metcalfe‐Roach et al. (2021)) and Agarwal et al. (2018) observed a greater adherence to the MIND diet to be more protective against PD than the MeDi.

Several studies on PD and dietary patterns have been performed, but none of them studied the association between the severity of PD and dietary patterns. Just Paknahad et al., in an intervention study, examined the severity of PD with a MeDi. Therefore, due to the limitations of studies in this field and the importance of the role of nutrition in the prevention and reduction of PD severity, we conducted this study to investigate the association of PD severity with the DASH, MeDi, and MIND diets.

## 2 METHODS

### 2.1 Study population

This comparative cross‐sectional study was conducted on 120 Parkinson's patients and 50 healthy people in Isfahan City, Iran, using a convenience sampling method. Samples were recruited from a clinical department, and the PD was diagnosed by a neurologist. People diagnosed with PD were checked according to the inclusion criteria, and then the data collection process was carried out. Inclusion criteria were age 40–80 years and confirmation of PD by a neurologist based on the Hoehn and Yahr (H & Y) Staging Scale (Emre et al., 2007). Exclusion criteria were receiving enteral and parenteral nutritional support and admission to hospital wards. The inclusion and exclusion criteria of the control group were similar to those of the PD group, except that they do not have any diseases. 50 healthy people were selected from the same city of Isfahan and from the general population. Participants in 2 groups were matched by age and gender.

Informed consent was obtained from each participant. The study was approved by the ethics committee of Tabriz University of Medical Sciences (IR.TBZMED.REC.1400.076) (https://ethics.research.ac.ir/ProposalCertificateEn.php?id=190612&Print).

### 2.2 Assessment of dietary intake

The dietary intake of participants was assessed using a 147‐item semi‐quantitative food frequency questionnaire (FFQ). All of the questionnaires were completed by a nutritionist. The FFQ contained a list of foods with serving sizes that are commonly consumed by Iranians. Participants were asked to report the frequency and quantity of each food item consumed in the past year on a daily, weekly, or monthly basis. The reliability and validity of the Persian version of this questionnaire have already been confirmed (Mirmiran et al., 2010). The modified Nutritionist IV software (version 1.0) was used to obtain the nutrient intake.

#### 2.2.1 Assessment of the DASH dietary score

To investigate participants' adherence to the DASH‐style diet, the DASH score was estimated based on foods and nutrients emphasized or minimized in the DASH dietary pattern, focusing on eight components: high intake of fruits, vegetables, nuts and legumes, dairy products, whole grains, and low intakes of sugar‐sweetened beverages and sweets, sodium, and red and processed meats. Then, subjects were categorized into quartiles of their energy‐adjusted intakes of foods and nutrients. Individuals in the highest quartile of fruits, vegetables, dairy products, nuts, and legumes were given a score of 4, and those in the lowest quartile were given a score of 1. With regard to the consumption of red and processed meat, sugar‐sweetened beverages and sweets, and sodium, we did vice versa: those with the highest consumption were given a score of 1, and those with the lowest consumption were given a score of 4. Finally, the DASH score for each participant was calculated by summing up the scores of eight components. The lowest and highest DASH scores could be 8 and 32, respectively.

#### 2.2.2 Assessment of MeDi dietary score

Based on the methodology of Panagiotakos et al. (Panagiotakos et al., 2004), MeDi dietary score was calculated focusing on 10 components, including fruits, vegetables, legumes, olive oil, fish, whole grains, and potatoes. For these items, with their high consumption, a score of 5, and for low consumption, a score of zero, is awarded. A score of 5 is considered for not consuming red and processed meat, poultry, and high‐fat dairy. In this method, each item is scored from 0 to 5 using a quintile, and the range of the total score is 0 to 50.

#### 2.2.3 Assessment of MIND dietary score

In the original scoring of the MIND diet, 15 dietary parameters were considered. Of these, 10 are known as brain‐healthy food groups (green leafy vegetables, other vegetables, nuts, berries, beans, whole grains, fish, poultry, olive oil, and wine), and 5 are known as brain‐unhealthy food groups (red meats, butter and stick margarine, cheese, pastries and sweets, and fried/fast food) (Morris, Tangney, Wang, Sacks, Barnes, et al., 2015; Morris, Tangney, Wang, Sacks, Bennett, & Aggarwal, 2015). In the current study, wine consumption was not considered in the score calculation because wine consumption was not common in the studied population. The other 14 food groups were used in the MIND diet construction. Participants were first classified based on the tertile cutoff points of their intakes of these 14 food components. Individuals in the lowest tertile of brain‐healthy food groups were given a score of 0, those in the middle tertile were given a score of 0.5, and those in the highest tertile were given a score of 1. With regard to brain‐unhealthy food groups, we did vice versa: individuals in the lowest tertile were given a score of 1 and those in the highest tertile were given a score of 0. Individuals in the middle tertile of these components were assigned a score of 0.5. Then, the overall MIND diet score was calculated by adding up all the dietary component scores. Therefore, each participant had a score between 0 and 14. This scoring was based on the method of the Morris, Tangney, Wang, Sacks, Barnes, et al. (2015) and Morris, Tangney, Wang, Sacks, Bennett, and Aggarwal (2015).

### 2.3 Assessment of anthropometric parameters

A tape meter was used to measure height in a standing position without shoes to the nearest 0.5 cm. The weight was measured using a SECA scale with minimal coverage and without shoes, with an accuracy of 100 grams. BMI was calculated by dividing weight (kg) by height squared (m).

### 2.4 Assessment of physical activity

The physical activity of the participants was assessed using the International Physical Activity Questionnaire (IPAQ; shortened version, 7‐day recall) (Craig et al., 2003). This survey evaluates walking, moderate‐intensity, and vigorous‐intensity physical activity profiles. The metabolic equivalent of task (MET)‐min/week was used to measure physical activity. According to the IPAQ, the total MET‐min/week score was classified into three levels of low, moderate, and high physical activity.

### 2.5 Severity of PD

The severity of PD was assessed by an expert neurologist. All aspects of PD were evaluated by the Movement Disorder Society‐sponsored Unified Parkinson's Disease Rating Scale (MDS‐UPDRS). MDS‐UPDRS includes 65 items and a range of 0 to 272 scores in 4 parts:

Part I, non‐motor aspects of experiences of daily living (13 items); Part II, motor aspects of experiences of daily living (13 items); Part III, motor examination (33 items); and Part IV, motor complications (6 items).

### 2.6 Statistical analysis

Findings from the study were analyzed using SPSS software version 22. Descriptive evidence and the Kolmogorov–Smirnov test were used to investigate the distribution of variables (normal, non‐normal). An independent *t*‐test was used to compare normal data, and a Mann–Whitney test was used for non‐normal data between the two studied groups. Linear regression was used to examine the association between variables, and binary logistics was used to determine the odds ratio. To consider the role of confounding factors, the analysis was performed in 2 models: multivariate model 1 (age, gender, BMI, and total calorie intake) and multivariate model 2 (multivariate model 1 plus smoking, diabetes, hypertension, thyroid disorder, cardiovascular diseases, medications, and physical activity).

The present study had some limitations. The effect of ingredients on dietary patterns was not investigated in this study. As well, data extraction of FFQ was done by gram/day, and the consumed load was not extracted. The small sample size was another limitation of this study. To eliminate selection bias in the sample, people were randomly selected after visiting the neurology clinic and confirming PD after reviewing the inclusion criteria.

## 3 RESULTS

### 3.1 Study population

Table 1 presents the characteristics of the participants. 65.8% of patients and 66% of healthy participants were men. There was no significant difference between the two groups in age, sex, and BMI (*p* > .05). The mean ± SD of total UPDRS was 46.2 ± 25.2 in the patient's group. Smoking status was significantly lower in patients (9.2%) compared with healthy subjects (24.0%) (*p* = .010). The number of people with high physical activity was higher in healthy subjects (12.0%) compared with patients (3.3%) (*p* = .030).

**TABLE 1:** Demographic characteristics of participants in the patient and healthy groups.

Variable	Participants		*p*‐Value
	Patients (*n* = 120)	Healthy (*n* = 50)	
Age^a^	60.8 ± 9.8	60.4 ± 9.8	.928
Gender^b^			.983
Men	79 (65.8)	33 (66)	
Women	41 (34.2)	17 (34)	
BMI^a^	25.3 ± 4.3	26.0 ± 5.0	.345
Comorbidities^b^			
Diabetes mellitus	10 (8.3)	–	
Hypertension	29 (24.2)	–	–
Thyroid disorders	14 (11.7)	–	–
Cardiovascular disease	15 (12.5)	–	–
Physical activity^b^			
Low	61 (50.8)	22 (44.0)	.802
Moderate	55 (45.8)	22 (44.0)	.962
High	4 (3.3)	6 (12.0)	**.030**
Smoking^b^ (%)	11 (9.2)	12 (24.0)	**.010**
Total UPDRS^a^	46.2 ± 25.2	–	–
Symptoms of non‐motor aspects of experiences of daily living^a^	7 ± 5.4	–	–
Symptoms of motor aspects of experiences of daily living^a^	12 ± 7.2	–	–
Symptoms of motor examination^a^	26 ± 15.6	–	–

^a^ Data are presented as Mean ± SD.

^b^ Data are presented as frequency (percent).

Abbreviation: BMI, body mass index.

The *p*‐value less than .05 is statistically significant.

As shown in Table 2, the mean score of the DASH dietary pattern was significantly lower in the PD group compared to the healthy group (*p* = .006). However, the mean score of MeDi and MIND dietary patterns did not significantly differ between the two groups (*p* > .05). In a comparison of the genders between the PD and healthy groups, the mean score of the DASH diet was significantly lower in men than in women in the healthy group (*p* = .018). However, there was no significant difference between men and women in the MeDi and MIND diets in the PD and healthy groups (*p* > .05) (Table 3).

**TABLE 2:** Characteristics of dietary patterns of participants in the patient and healthy groups.

Variables	Participants		*p*‐Value
	Patient (*n* = 120)	Healthy (*n* = 50)	
DASH score (Mean ± SD)	19.6 ± 3.8	20.9 ± 2.3	**.006**
MeDi score (Mean ± SD)	29.9 ± 4.8	30.2 ± 4.4	.762
MIND score (Mean ± SD)	7.0 ± 1.7	6.6 ± 1.3	.257

Abbreviations: DASH, dietary approach to stop hypertension; MeDi, Mediterranean diet; MIND, Mediterranean‐DASH intervention for neurodegenerative delay.

The *p*‐value less than .05 is statistically significant.

**TABLE 3:** Characteristics of dietary patterns of participants in the patient and healthy groups based on gender.

Variable	Participants					
	Patient (*n* = 120)			Healthy (*n* = 50)		
	Men	Women	*p*‐Value	Men	Women	*p*‐Value
DASH score	19.2 ± 3.6	20.4 ± 4.0	.119	20.4 ± 2.1	22.1 ± 2.5	**<.018**
MeDi score	29.8 ± 4.7	30.3 ± 5.0	.581	30.9 ± 3.9	28.8 ± 4.9	.149
MIND score	6.9 ± 1.6	7.2 ± 1.8	.565	6.6 ± 1.1	6.7 ± 1.6	.718

*Note*: Data are presented as Mean ± SD.

Abbreviations: DASH, dietary approach to stop hypertension; MeDi, Mediterranean diet; MIND, Mediterranean‐DASH intervention for neurodegenerative delay.

The *p*‐value less than .05 is statistically significant.

### 3.2 Association between the severity of PD and dietary patterns

As shown in Table 4, there was no significant association between the dietary patterns of DASH, MeDi, and MIND and the severity of PD (*p* > .05). As well, there was no association between the dietary patterns and severity of PD based on parts I (non‐motor aspects of experiences of daily living), II (motor aspects of experiences of daily living), and III (motor examination) of the MDS‐UPDRS (Table 5).

**TABLE 4:** Association of Parkinson's disease severity with DASH, MeDi, and MIND diets.

Variable	Univariate model		Multivariate model 1		Multivariate model 2	
	B (95% CI)	*p*‐Value	*B* (95% CI)	*p*‐Value	*B* (95% CI)	*p*‐Value
DASH	−0.17 (−1.39, 1.05)	.78	−0.27 (−1.53, 0.98)	.67	−0.29 (−1.58, 1.00)	.66
MeDi	0.13 (−0.67, 0.93)	.74	−0.03 (−0.86, 0.79)	.94	−0.01 (−0.83, 0.85)	.98
MIND	1.86 (−0.86, 4.58)	.18	1.64 (−1.23, 4.51)	.26	1.71 (−1.25, 4.68)	.25

*Note*: Model 1: Variables included in the analysis were age, sex, BMI, and caloric intake. Model 2: Variables included in the analysis were variables included in model 1 plus smoking, diabetes, hypertension, thyroid disorder, cardiovascular diseases, medications, and physical activity.

Abbreviations: DASH, dietary approach to stop hypertension; MeDi, Mediterranean diet; MIND, Mediterranean‐DASH intervention for neurodegenerative delay.

**TABLE 5:** Association of DASH, MeDi, and MIND diets with the severity of PD based on parts I, II, and III of the UPDRS.

Variable	Non‐motor aspects of experiences of daily living		Motor aspects of experiences of daily living		Motor examination	
	*B* (95% CI)	*p*‐Value	*B* (95% CI)	*p*‐Value	*B* (95% CI)	*p*‐Value
DASH						
Univariate model	0.17 (−0.09, 0.43)	.20	−0.04 (−0.38, 0.31)	.84	−0.31 (−1.06, 0.44)	.42
Multivariate model 1	0.08 (−0.18, 0.34)	.53	−0.05 (−0.41, 0.30)	.77	−0.32 (−1.09, 0.45)	.41
Multivariate model 2	0.09 (−.18, 0.35)	.51	−0.07 (−0.43, 0.29)	.71	−0.33 (−1.12, 0.46)	.41
MeDi						
Univariate model	0.03 (−0.14, 0.20)	.74	0.09 (−0.14, 0.32)	.44	−0.03 (−0.53, 0.46)	.90
Multivariate model 1	−0.04 (−0.21, 0.13)	.64	0.10 (−0.14, 0.33)	.41	−0.13 (−0.63, 0.38)	.62
Multivariate model 2	−0.03 (−0.20, 0.14)	.74	0.10 (−0.13, 0.33)	.39	−0.10 (−0.61, 0.42)	.71
MIND						
Univariate model	0.57 (−0.01, 1.15)	.05	0.23 (−0.54, 1.01)	.55	0.74 (−0.95, 2.42)	.39
Multivariate model 1	0.37 (−0.23, 0.96)	.22	0.36 (−0.46, 1.17)	.39	0.59 (−1.19, 2.36)	.51
Multivariate model 2	0.39 (−0.21, 1.00)	.20	0.32 (−0.51, 1.15)	.44	0.68 (−1.15, 2.52)	.46

*Note*: Model 1: Variables included in the analysis were age, sex, BMI, and caloric intake. Model 2: Variables included in the analysis were variables included in model 1 plus smoking, diabetes, hypertension, thyroid disorder, cardiovascular diseases, medications, and physical activity.

Abbreviations: DASH, dietary approach to stop hypertension; MeDi, Mediterranean diet; MIND, Mediterranean‐DASH intervention for neurodegenerative delay.

### 3.3 Association between the risk of PD and dietary patterns

As shown in Table 6, PD patients adhere less to the DASH dietary pattern by 15% (OR = 0.856, 95% CI: 0.751–0.976, *p* = .020). PD patients in quartiles 3 and 4 of the DASH dietary pattern were lower by 86% (OR = 0.140, 95% CI: 0.038–0.520, *p* = .003) and 87% (OR = 0.138, 95% CI: 0.033–0.576, *p* = .007), respectively, than quartiles 1 (Table 7).

**TABLE 6:** Association of DASH, MeDi, and MIND dietary patterns with the risk of Parkinson's disease in a continuous model.

Variables	Univariate model		Multivariate model 1		Multivariate model 2	
	Odds ratio (95% CI)	*p*‐Value	Odds ratio (95% CI)	*p*‐Value	Odds ratio (95% CI)	*p*‐Value
DASH	**0.893 (0.809**. **0.986)**	**.025**	**0.893 (0.805**. **0.990)**	**.031**	**0.856 (0.751**. **0.976)**	**.020**
MeDi	0.989 (0.921, 1.063)	.768	0.996 (0.924, 1.073)	.918	0.987 (0.903, 1.080)	.781
MIND	1.155 (0.935, 1.426)	.182	1.205 (0.963, 1.507)	.102	1.240 (0.931, 1.652)	.142

*Note*: Model 1: Variables included in the analysis were age, sex, BMI, and caloric intake. Model 2: Variables included in the analysis were variables included in model 1 plus smoking, diabetes, hypertension, thyroid disorder, cardiovascular diseases, medications, and physical activity.

Abbreviations: DASH, dietary approach to stop hypertension; MeDi, Mediterranean diet; MIND, Mediterranean‐DASH intervention for neurodegenerative delay diet.

The *p*‐value less than .05 is statistically significant.

**TABLE 7:** Association of the DASH, MeDi, and MIND diets with the risk of Parkinson's disease based on quartiles.

	Q1	Q2		Q3		Q4	
		OR (95% CI)	*p*‐Value	OR (95% CI)	*p*‐Value	OR (95% CI)	*p*‐Value
DASH							
Univariate model	Ref	0.309 (0.087, 1.092)	.068	0.171 (0.053, 0.551)	.003	0.204 (0.060, 0.694)	.011
Multivariate model 1	Ref	0.317 (0.089, 1.137)	.078	0.170 (0.052, 0.555)	.003	0.204 (0.057, 0.721)	.014
Multivariate model 2	Ref	**0.216 (0.051**. **0.923)**	**.039**	**0.140 (0.038**. **0.520)**	**.003**	**0.138 (0.033**. **0.576)**	**.007**
MeDi							
Univariate model	Ref	0.822 (0.316, 2.141)	.688	0.531 (0.195, 1.441)	.214	1.142 (0.405, 3.222)	.801
Multivariate model 1	Ref	0.855 (0.323, 2.260)	.752	0.543 (0.189, 1.561)	.257	1.246 (0.425, 3.655)	.689
Multivariate model 2	Ref	1.156 (0.372, 3.587)	.802	0.434 (0.117, 1.604)	.211	1.392 (0.392, 4.947)	.609
MIND							
Univariate model	Ref	1.400 (0.549, 3.570)	.481	1.280 (0.517, 3.170)	.594	1.848 (0.705, 4.844)	.212
Multivariate model 1	Ref	1.399 (0.540, 3.623)	.489	1.486 (0.574, 3.849)	.415	2.161 (0.796, 5.870)	.131
Multivariate model 2	Ref	1.216 (0.397, 3.722)	.732	1.758 (0.601, 5.148)	.303	1.594 (0.495, 5.130)	.434

*Note*: Model 1: Variables included in the analysis were age, sex, BMI, and caloric intake. Model 2: Variables included in the analysis were variables included in model 1 plus smoking, diabetes, hypertension, thyroid disorder, cardiovascular diseases, medications, and physical activity.

Abbreviations: DASH, dietary approach to stop hypertension; MeDi, Mediterranean diet; MIND, Mediterranean‐DASH intervention for neurodegenerative delay diet; CI, confidence interval.

The *p*‐value less than .05 is statistically significant.

No significant association was observed between adherence to the MeDi (OR = 0.987, 95% CI: 0.903–1.080, *p* = .781) and MIND (OR = 1.240, 95% CI: 0.931–1.652, *p* = .142) dietary patterns and the risk of PD (*p* > .05) (Table 6).

## 4 DISCUSSION

In the present study, high adherence to the DASH dietary pattern was inversely associated with the risk of PD, after adjustment for potential confounding factors. In contrast, neither the MeDi nor MIND dietary patterns were associated with the risk of PD. No significant association was observed between DASH, MeDi, and MIND dietary patterns and the severity of the disease.

To our knowledge, this is the first study to investigate the association between dietary patterns and the severity and risk of PD. The present study indicates that increasing adherence to the DASH dietary pattern decreased the risk of PD by 15%. Also, in the ranking of DASH scores, it was shown that adherence to the DASH dietary pattern in quartile 4 reduced the risk of PD by 87%. According to the results of this study, the MeDi and MIND dietary patterns were not associated with the risk of PD. This result is probably related to patient life style, genetic factors, and environmental conditions. Evidence for an association between dietary patterns and the severity of PD is limited. Only one intervention study has been done regarding this association. Paknahad et al. investigated the effect of MeDi on the severity of PD (Paknahad et al., 2022). In this study, 70 participants with PD were enrolled and divided into two groups. One group followed MeDi, and another followed the Iranian traditional diet. The results of this study, contrary to the results of the present study, reported the positive effect of MeDi on the severity of PD. The adherence duration of MeDi in the study by Paknahad et al. was 10 weeks. For the MeDi group in this study, the dietary plan was prepared based on the recommendations of the MeDi for each person. Also, the small number of participants and the difference in the type of study could be the reasons for the difference in the results of this study.

Gao et al. (Gao et al., 2007) examined the association between dietary patterns and the risk of PD. The study found that dietary patterns with a high intake of fruits, vegetables, legumes, whole grains, nuts, fish, and poultry, low saturated fatty acids, and moderate alcohol consumption may protect against PD. These food groups are part of the DASH dietary pattern. In the present study, it was found that high adherence to the DASH dietary pattern may decrease the risk of PD, and this result was linked with the Gao et al. study. Contrary to our results, Alcalay et al. (Alcalay et al., 2012) reported that adhering to MeDi in PD patients was lower than in the healthy group. This result could be due to the large number of participants in the study. The study results of Agarwal et al. (2018) indicated that the MIND dietary pattern was associated with decreased risk and progression of PD, contrary to the present study. This result could be due to the use of cohort data.

Oxidative stress and inflammation are two major causes of neurodegenerative diseases such as PD. Alpha‐synuclein aggregation by oxidative stress, activation of microglial cells, and degeneration of dopaminergic neurons with inflammation promote PD (Tiwari & Pal, 2017). On the other hand, the DASH dietary pattern could decrease malondialdehyde (MDA) levels and increase reduced glutathione (GSH) levels significantly (Pirouzeh et al., 2020). According to the literature, MDA is the most mutagenic product of the reaction of unsaturated fatty acids with oxygen (Ayala et al., 2014). As well, MDA is the best indicator of oxidative stress (Gaweł et al., 2004). Polyphenols in the components of the DASH dietary pattern can reduce MDA and control oxidative stress (Alvarez‐Suarez et al., 2011). The anti‐inflammatory mechanism of the Dash dietary pattern is unknown. Dietary fiber may increase the microbial species in the colon that produce anti‐inflammatory cytokines (Poullis et al., 2004). Also, the DASH dietary pattern significantly reduced hs‐CRP levels (Saneei et al., 2014). Compounds of the DASH dietary pattern such as fruits, vitamin C, dietary fiber, and low‐fat dairy might be the reason for this beneficial effect on hs‐CRP levels (Esmaillzadeh et al., 2006; Esmaillzadeh & Azadbakht, 2010; North et al., 2009; Wannamethee et al., 2006). Therefore, the DASH dietary pattern, by reducing inflammation and then preventing microglial cell activation and degeneration of dopaminergic neurons, may decrease the risk of PD. The DASH dietary pattern recommends consumption of vegetables, fruits, low‐fat dairy products, whole grains, poultry, fish, and nuts and reduce consumption of red meat, sweets, sweet drinks, total fat, and cholesterol. Adherence to this healthy dietary pattern, in addition to reducing the risk of PD disease, prevents many other diseases, and helps to increase public health.

## 5 CONCLUSION

The findings indicate that high adherence to the DASH dietary pattern may protect against PD. However, there was no association between MeDi and MIND dietary patterns with the risk of PD and severity of PD. An investigation of the DASH dietary pattern effect on PD is suggested by interventional studies. It is also necessary to study the effects of dietary pattern ingredients on the risk and severity of PD in future studies.

## AUTHOR CONTRIBUTIONS

**Majid Keramati:** Conceptualization (equal); data curation (equal); formal analysis (equal); investigation (equal); project administration (equal); software (equal); writing – original draft (equal). **Sorayya Kheirouri:** Conceptualization (equal); methodology (equal); project administration (equal); supervision (equal); validation (equal); writing – review and editing (equal). **Masoud Etemadifar:** Data curation (equal); investigation (equal); methodology (equal); supervision (equal).

## FUNDING INFORMATION

This study was funded by the Vice‐chancellor for Research and Student Research Committee of Tabriz University of Medical Sciences, Tabriz, Iran (grant number: 66548).

## CONFLICT OF INTEREST STATEMENT

The authors declare that they do not have any conflicts of interest.

## ETHICS STATEMENT

This study was approved by the ethics committee, Tabriz University of Medical Sciences (IR.TBZMED.REC.1400.076) (https://ethics.research.ac.ir/ProposalCertificateEn.php?id=190612&Print).

## INFORMED CONSENT

Written informed consent was obtained from all study participants.

## Data Availability

The data that support the findings of this study are available from the corresponding author upon reasonable request.
